# Event and Comment

**Published:** 1907-09-15

**Authors:** 


THE
### DENTAL REGISTER.

Vol. LXI. August 15, 1907. No. 8

### EVENT AND COMMENT.

### Independent Dental Journalism.

The National Dental Association has at last decided to take up the much discussed proposition of having a dental Journal that shall represent the best ideal in professionalism. We have had a number of so called independent journals started and maintained at great expense of money, and lots of wear and tear of mind and muscle on the part of self sacrificing patriots. In due time they have gone their ways and we doubt very much if they have been worth the effort and the hard feelings such protests usually cause. It may be that by this form of agitation we trade journalists are kept up to a higher standard of professionalism and are saved from retrograding into mere trades papers. If these independent journals have done any good that is commensurate with their cost we should not deplore their existence. Possibly we should rejoice now that our great representative national organization is about to test out in the most favorable manner the question as to whether the so called "trade journal" or the strictly professional journal shall best meet the needs of our profession. We are not acquainted with the plans of the new national Dental Journal, but no doubt it will embody all the excellent features of the present trade journals in its general make up; and in addition it will eradicate a professional influence that shall transform the charlatans and tyros of our profession into the most humble servants known to all times and callings.

The July *Items of Interest* contains a paper by a young man who never edited a trade dental journal we are certain, or he could not seriously make such unwarranted attacks on the only literary output our profession has had that it could depend upon. The discussion that followed the reading of the paper showed conclusively that there is no serious sympathy with such an unjust attack on our dental periodicals, and anyone who knows how the editorial work of our present journals is conducted will feel disgusted that we can not discuss such vital issue in a more magnanimous spirit. We sincerely trust that the new editor of the Journal of the National Dental Association shall be broad enough to take high professional ground in his treatment of this and kindred subjects. There is plenty of room for this new enterprise and we gladly welcome it and shall do all we can to sustain it, by courteous and professional treatment and such financial aid as may fall to us. The Dental Register has lived longer than any other dental publication, not because it has been the "fittest," but because it has always been the policy of its editors to represent the profession in a dignified and useful way. It has never entered into commercial competition with other journals, but has steadily tried to gather together for permanent record and distribution to its constituents the best attainments of the profession in general and of the locality where it is printed particularly. It has made many blunders and fallen far short in many undertakings, but its career has been an honest purpose always to do the best it could for the advancement of the profession's higher interests. We honestly believe this can be said of the other so called trade journals. Some have accomplished vastly more than we, because their circumstances and opportunities have been greater, but all alike have done what they could for the betterment of conditions as they meet them from month to month, year in and year out. It takes time to educate an editor, just as it does to bring a fruit orchard into full bearing, and the editor who starts out to reform everything in sight, will soon find that he has little or no influence and the profession will get very tired of his journal and he will retire disappointed and permanently agrieved. Therefore we sincerely trust the new Journal may not be wholly a protest, but a means of recording and distributing more widely than any other journal has or can the invaluable material which the active minds and hands of our progressive science and art is producing in quantity and quality never before approached in the history of the profession. We have none too many journals, and one more if it is good enough to excel will live and prosper, and should be a potent instrument in the future developement of our science and art.

